# Commentary: It will be worth it, in the end: a model of naturalistic intertemporal choice

**DOI:** 10.3389/fpsyg.2026.1869082

**Published:** 2026-08-26

**Authors:** Kenneth R. Paap

**Affiliations:** Department of Psychology, San Francisco State University, San Francisco, CA, United States

**Keywords:** activation dynamics, attention, intertemporal choice, preference reversals, temporal discounting

## Introduction

Newall and Peacey (2026) propose an important reframing of intertemporal choice. Rather than treating temporal discounting as a primitive feature of valuation, their model explains intertemporal failures as a consequence of bounded future-oriented cognition. Decision makers construct value by mentally simulating future outcomes, but this simulation extends only to a finite attentional horizon. Outcomes beyond that horizon exert little influence on choice, yielding preference reversals and systematic failures such as addiction, procrastination, and smaller-sooner choice. Extending the horizon reduces these errors and aligns behavior more closely with long-term preferences. The formalization of a finite horizon (S), the taxonomy of error types, and the integration with episodic future-thinking interventions together provide a clear and testable account of naturalistic intertemporal choice.

Newall and Peacey (2026) also cite the Comparison with Goal States Model (CGSM; Suri and Paap, 2024), although the relationship between the two accounts is not developed. The present commentary reinterprets their central claim—that intertemporal choice depends on how far into the future a person mentally simulates—as a constraint on the temporal propagation of goal-based control. This process-level account specifies how a finite attentional horizon can produce the behavioral regularities identified in their model.

## From computational constraint to process-level mechanism

Newall and Peacey (2026) specify the computational constraint governing choice: utilities beyond a truncated simulation horizon receive no effective weight. What remains open is how this constraint is implemented by the processes that generate a choice. The CGSM provides a concrete process-level implementation. In that model, options compete through activation dynamics shaped by goal representations. Goal-consistent options are amplified while competing alternatives are suppressed, and these dynamics unfold over time. When a decision is made early in the settling process, immediately rewarding options are more likely to dominate; when processing continues, goal-consistent, often longer-term outcomes exert greater influence.

On this interpretation, the attentional horizon (S) is the effective duration over which goal-related activation is allowed to propagate. Truncated simulation corresponds to terminating this dynamic process before goal influence has fully shaped the competitive landscape. Thus, the horizon need not be treated only as a boundary on represented future utility; it can also be understood as a boundary on the time available for goal-directed control to alter the state of the decision system.

## Reinterpreting simulation

This interpretation also changes the status of “mental simulation.” Rather than a separate process appended to decision making, simulation may be the subjective experience of extended goal-modulated settling. What Newall and Peacey (2026) describe as “thinking further into the future” can be understood as allowing goal-relevant information more time to influence choice. The integration yields three related claims:

- Preference reversals occur when a choice is committed before goal influence has propagated sufficiently.
- Extending the horizon corresponds to prolonging the temporal window of control.
- Future-oriented interventions may work by increasing the duration or strength of goal influence rather than by changing underlying preferences.

Temporal discounting then emerges not because future value is intrinsically reduced, but because the decision system settles before those values are fully integrated.

## Theoretical and empirical implications

This interpretation connects Newall and Peacey's (2026) model to computational accounts in which cognitive control is allocated according to the expected value of control and the interaction of motivation, effort, and goal-directed processing (Botvinick and Braver, 2015; Shenhav et al., 2013). These accounts do not treat control as a latent store that is simply drawn down; they emphasize how the intensity and duration of control are determined within an ongoing decision process. CGSM adds a specific activation-based realization of that general idea.

The integration also generates tests that go beyond either account alone. If the attentional horizon reflects the duration of goal-based control dynamics, manipulations that increase processing time without explicitly encouraging future thought—such as response delays or enforced deliberation—should reduce intertemporal errors in a manner similar to episodic future-thinking interventions. Interventions that strengthen goal representations should improve long-term choice even when simulation depth is unchanged, by accelerating the propagation of goal-consistent activation. Process measures, including response-time distributions, mouse trajectories, or sequential-sampling signatures, should show that long-term choices are associated with more extended or stable settling dynamics.

These predictions distinguish two interpretations. If future simulation is a distinct cognitive operation, explicit simulation manipulations should produce benefits beyond those achieved by additional processing time alone. If both manipulations operate by extending control dynamics, their effects should be functionally interchangeable. Likewise, improvement in choice should track the effective influence of goal representations more closely than self-reported simulation depth.

## Conclusion

Newall and Peacey's (2026) model offers a compelling alternative to traditional discounting accounts by locating intertemporal failures in limits on future-oriented cognition. Interpreting the attentional horizon as a constraint on the temporal propagation of goal-based control provides a bridge from computational description to process-level mechanism. Temporal discounting, future simulation, and self-control can then be understood as different manifestations of a single capacity-limited control system operating over time. On this view, what appears as discounting is not a property of value, but a consequence of when the decision process is terminated.
